# Patient-Specific Variations in Biomarkers across Gingivitis and Periodontitis

**DOI:** 10.1371/journal.pone.0136792

**Published:** 2015-09-25

**Authors:** Radhakrishnan Nagarajan, Craig S. Miller, Dolph Dawson, Mohanad Al-Sabbagh, Jeffrey L. Ebersole

**Affiliations:** 1 Division of Biomedical Informatics, Department of Biostatistics, College of Public Health, University of Kentucky, Lexington, KY, United States of America; 2 Center for Oral Health Research, College of Dentistry, University of Kentucky, Lexington, KY, United States of America; 3 Division of Oral Medicine, Department of Oral Health Practice, College of Dentistry, University of Kentucky, Lexington, KY, United States of America; 4 Division of Periodontics, Department of Oral Health Practice, College of Dentistry, University of Kentucky, Lexington, KY, United States of America; University of Florida, UNITED STATES

## Abstract

This study investigates the use of saliva, as an emerging diagnostic fluid in conjunction with classification techniques to discern biological heterogeneity in clinically labelled gingivitis and periodontitis subjects (80 subjects; 40/group) A battery of classification techniques were investigated as traditional single classifier systems as well as within a novel selective voting ensemble classification approach (SVA) framework. Unlike traditional single classifiers, SVA is shown to reveal patient-specific variations within disease groups, which may be important for identifying proclivity to disease progression or disease stability. Salivary expression profiles of IL-1ß, IL-6, MMP-8, and MIP-1α from 80 patients were analyzed using four classification algorithms (LDA: Linear Discriminant Analysis [LDA], Quadratic Discriminant Analysis [QDA], Naïve Bayes Classifier [NBC] and Support Vector Machines [SVM]) as traditional single classifiers and within the SVA framework (SVA-LDA, SVA-QDA, SVA-NB and SVA-SVM). Our findings demonstrate that performance measures (sensitivity, specificity and accuracy) of traditional classification as single classifier were comparable to that of the SVA counterparts using clinical labels of the samples as ground truth. However, unlike traditional single classifier approaches, the normalized ensemble vote-counts from SVA revealed varying proclivity of the subjects for each of the disease groups. More importantly, the SVA identified a subset of gingivitis and periodontitis samples that demonstrated a biological proclivity commensurate with the other clinical group. This subset was confirmed across SVA-LDA, SVA-QDA, SVA-NB and SVA-SVM. Heatmap visualization of their ensemble sets revealed lack of consensus between these subsets and the rest of the samples within the respective disease groups indicating the unique nature of the patients in these subsets. While the source of variation is not known, the results presented clearly elucidate the need for novel approaches that accommodate inherent heterogeneity and personalized variations within disease groups in diagnostic characterization. The proposed approach falls within the scope of P4 medicine (predictive, preventive, personalized, and participatory) with the ability to identify unique patient profiles that may predict specific disease trajectories and targeted disease management.

## Introduction

Movement towards an era of personalized medicine and individualized clinical decisions in periodontology requires significant improvement in our ability to define risk and predict disease progression. While the medical field routinely makes clinical diagnoses based on signs and symptoms (*e*.*g*. pneumonia, diarrhea), decisions on patient management and treatment do not stop there. Modern medicine and its evolution towards P4 medicine (http://www.p4mi.org/) integrates clinical manifestations with biological assessments that enable the physician to focus on specific disease etiology and unique features of the patient in finalizing a treatment strategy. However, current practice in differential diagnosis and treatment options in dentistry often stops at the clinical and radiographic presentation of the patient, despite the expansion of information now available from biological fluids, plaque and tissue.

Gingivitis is a reversible condition associated with bacterial biofilms that generally resolves clinically in about one week after the reinstitution of oral hygiene procedures [1]. It is an often-overlooked disease, despite being the “gateway” to periodontitis for a significant portion of the population [2–5]. Gingivitis affects a large percentage of the U.S. population [6, 7] and is generally accepted that, if left untreated, may ultimately progress to periodontitis in a subset of individuals.[4, 8] In fact, a recent report by Lang et al. [2] focusing on gingivitis as a risk factor in periodontal disease identified that approximately 37% of teeth with consistent gingivitis progressed to periodontitis and tooth loss, while non-inflamed teeth showed a 99.5% survival. Additionally, the heterogeneity of these disease populations is evident in that various treatment studies of both gingivitis and periodontitis have shown that a portion of the gingivitis population does not respond well to standard mechanical therapy [9] nor do they return to biological health in a timely manner [10], and a portion of the periodontitis population remains somewhat refractory to treatment using standard approaches [9].

Differential host responses are the basis for various levels of susceptibility in chronic inflammatory diseases, such as periodontitis [11–13]. Engagement of host cell receptors with bacterial components activate immune and nonimmune cells to secrete an array of cytokines and inflammatory mediators, *eg*. interleukin(IL)-1ß, IL-6, and tumor necrosis factor (TNF)α, that results in production of matrix metalloproteinases (MMPs) that undermine the integrity of the gingival tissues[14]. These biomolecules of inflammation can be measured in oral fluids [15, 16], and saliva [17–19], supporting their potential for diagnostic utility regarding oral health and disease. This study selected 4 of these salivary analytes for evaluation based upon literature documenting their detection and likely role in periodontal disease pathology. IL-1ß has been identified in gingival crevicular fluid [GCF, [20]] and saliva [10, 21, 22] in elevated levels and has been related to disease extent/severity [23] and decreasing levels with successful therapy [24]. Increased IL-6 levels have also been shown in GCF [25] and saliva [26] in periodontitis, as well as in periodontitis tissues [27]. As with IL-1ß, treatment studies show decreased levels of IL-6 with improved clinical parameters [28]. MMP-8 (neutrophil collagenase) is increased at sites of inflammation in both GCF (20) and saliva [10, 21, 26] in periodontitis and has been suggested as a potential diagnostic molecule for the disease [16]. Finally, macrophage inflammatory protein (MIP)-1α (CCL3) is a chemokine produced by many cell types linked to inflammatory lesions [29, 30] and stimulates monocytes and/or osteoclast progenitor cells to become active osteoclasts [31]. Few studies are available regarding this biomolecule in saliva, although a single study indicated that MIP-1α is elevated in saliva of aggressive periodontitis patients [32] and we have shown increased concentrations in chronic periodontitis patients [33].

Recent studies have provided novel insights into changes in molecular signatures across the spectrum of periodontal disease [34–41]. Classification techniques have also been used for investigating the predictive potential of molecular markers in discerning distinct disease groups [36]. However, a majority of existing approaches have clear limitations: (a) Existing techniques traditionally do not accommodate or provide insights into potential patient-specific variations within a disease group while discerning distinct disease groups. (b) Pre-defined thresholds and filters are often used as pre-processing in eliminating patient samples that do not conform to a given criteria in an attempt to homogenize the samples within a disease group. While helpful in reducing the dimensionality, such filters can eliminate natural variations within a disease group. (c) They aggregate the expression profiles across samples within each group. However, these in turn may minimize differences between distinct disease groups, as well as prevent identifying potential subpopulations with unique characteristics in the groups. (d) More importantly, a majority of the existing approaches use the same set of fixed markers as representative of each sample within and between the groups of interest and use a single classifier in discerning the disease groups of interest. Ensemble approaches that use a battery of classifiers as opposed to a single classifier have been shown to perform better [42–46]. In this report, we use a selective voting ensemble classification approach (SVA) [47, 48] in conjunction with a battery of classification techniques and salivary biomarkers (IL-1ß, IL-6, MIP-1α, MMP-8) to discern clinically-labeled gingivitis and periodontitis groups. The four salivary biomarkers are targeted because they correspond to the underlying biological processes that occur during gingivitis and periodontitis (i.e., inflammation, tissue destruction and bone remodeling) [49, 50]. Thus, their use in conjunction with the SVA approach is hypothesized to reveal patient-specific variations within disease groups and potentially important for identifying an increased risk for disease progression.

## Materials and Methods

### Human Subjects and Clinical Evaluation

Case-control studies were conducted at the University of Kentucky, College of Dentistry from 2009 through 2013 with protocols approved by the Institutional Review Board. Participants were recruited from the clinic populations of the College of Dentistry. Each participant was given verbal and written information that described the nature of the study, and each signed informed consent prior to enrollment of the study. Inclusion criteria included subjects older than 18 years of age who were in good general health (excluding the case definition) and had a minimum of 20 teeth. BOP (bleeding on probing), PPD (probing pocket depth), and CAL (clinical attachment level) were measured using a full mouth periodontal examination at six locations per tooth (mesial-buccal, mid-buccal, distal-buccal, mesial lingual, mid-lingual, and distal-lingual). After the measurement of PPDs, all sites were observed for bleeding on probing BOP [51].

Two oral disease groups were analyzed. The 40 subjects in the gingivitis group (age: 27.5±4.5; 50% female) had BOP at ≥20% of sites (6 sites per tooth), <10% of sites with PPD ≥4 mm, and no sites [52] ≥2 mm. An equal number of subjects (n = 40) in the periodontitis group (age: 40.8±10.5; 43% female) had BOP at >20% of sites, with >10% of sites with PPD ≥4 mm and CAL ≥2 mm. Individuals were excluded from either group if there was evidence of general health issues, chronic inflammatory conditions, pregnancy, use of antibiotics or other mucosal inflammatory disease [53]. While smoking has been clearly identified as a risk factor in the extent and severity of periodontitis [54, 55] it was not included as a feature in the classification approach. Population demographics of the cohorts based on patient-reported information indicated none of the gingivitis patients to be smokers and ~27% of the periodontitis patients as smokers.

### Salivary Samples and Molecular Biomarkers

Whole unstimulated saliva was collected on ice between 9 AM and 3 PM from both groups prior to clinical evaluation as we have described previously [53, 56] according to a modification of the method described by Navazesh [57]. Aliquots were prepared and frozen at -80°C until analyzed. The MILLIPLEX MAP Kit (Billerica, MA, USA) was used to detect IL-1ß, IL-6, MMP-8, and MIP-1α as we have described previously [10].

#### Selective Voting Ensemble Classification (SVA)

**Fig 1:**
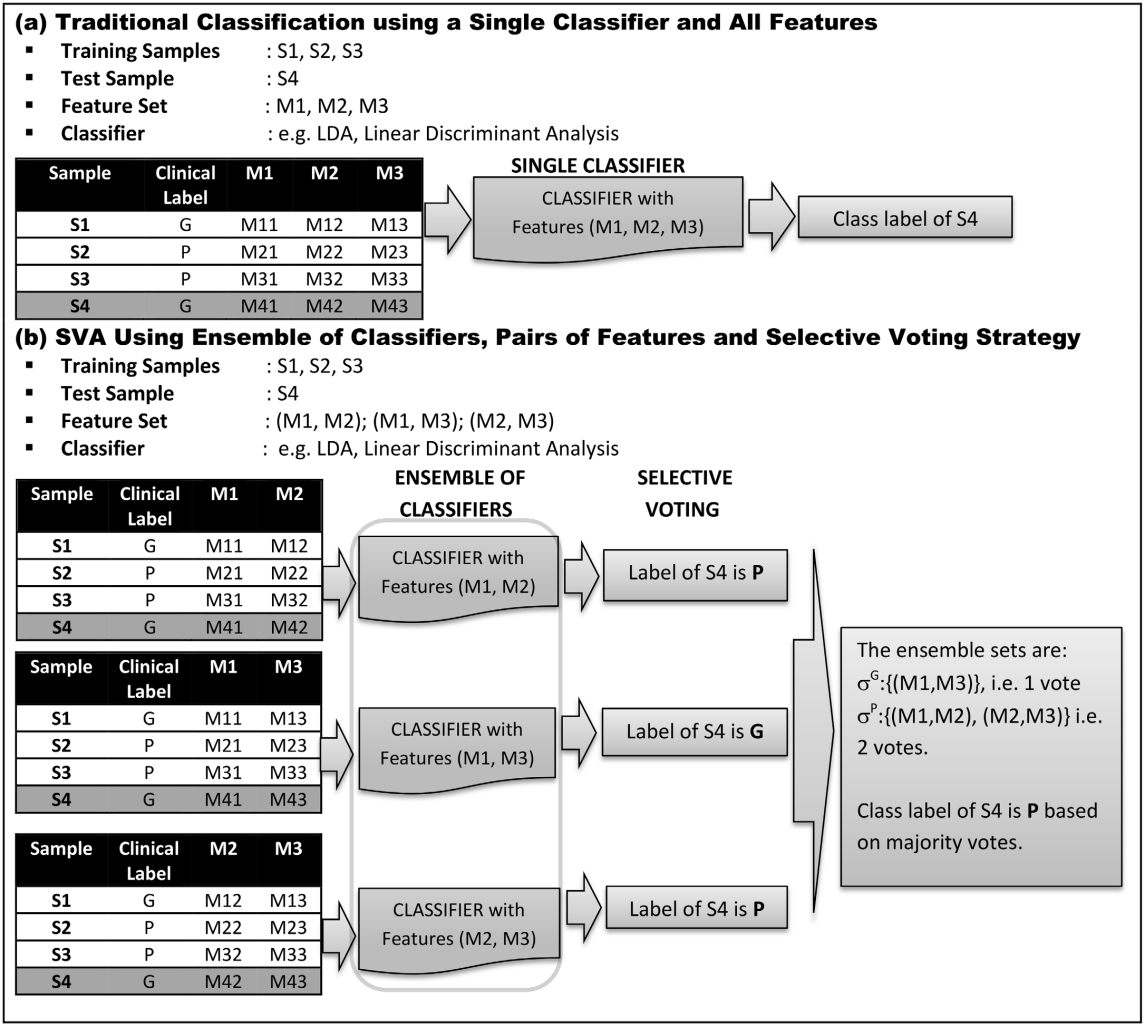
Working Principle of the Selective Voting Approach. An example of traditional binary classification using all features and a single classifier is shown in **(a)**. SVA approach that selectively votes on samples using an ensemble of classifiers and pairs of features is shown in **(b)**. Three molecular markers (M1, M2, M3) and four samples (S1, S2, S3, S4) with clinical labels (G, P, P, G) corresponding to gingivitis (G) and periodontitis (P) are considered. Sample S4 is set aside as the test sample with (S1, S2, S3) as the training samples in the classification process. The single classifier approach implicitly assumes the markers to be identical and fixed across the samples and votes the test sample as either G or P. In contrast, SVA enables the pairs of features to selectively vote on the samples. For the above example we have ^3^C_2_ = 3 potential classifiers {(M1, M2), (M1, M3), (M2, M3)}. While {(M1, M2), (M2, M3)} vote on the test sample as gingivitis represented by σ^G^ {(M1, M3)} vote on the test sample as periodontitis represented by σ^P^. Based on majority votes (2 votes as periodontitis and 1 vote as gingivitis) the SVA class label of the test sample is deemed as periodontitis. More importantly, the test sample with clinical label gingivitis is classified as periodontitis by SVA. It is important to note that if we were to repeat the above process by setting aside the other gingivitis sample (S1) as test sample, the ensemble sets and votes need not necessarily be the same. This critical selective voting aspect of SVA enables personalization of the ensemble sets across samples within and between the classes (i.e. disease groups). Also, in contrast to the single classifier that deems the given test sample as G or P, SVA determines the proclivity of the test sample to G and P given by the normalized vote counts (1/3, 2/3).

SVA approach for discerning gingivitis and periodontitis and extracting patient-specific profiles is detailed below. A comparison of the proposed SVA approach and traditional classifications is also depicted in Fig 1 for convenience.

#### Given

Salivary expression profiles of (*m*) genes across gingivitis (*n*_*gin*_) and periodontitis (*n*_*per*_) subjects represented by the matrix *X*_*mxn*_ where *n = n*_*gin*_ *+n*_*per*_. Salivary expression profiles were log-transformed and normalized to zero-mean and unit variance. Clinical labels of the *n* samples based on the three clinical parameters (BOP, PD, CAL) in *λ*_1xn_ where

$$\lambda \left(k\right)\;=\;-1\text{ implies the }k^{th}\text{ sample is gingivitis}$$

$$+1\text{ implies the }k^{th}\text{ sample is periodontitis}$$

Initialize the voting matrices across the samples as follows:

$$\upsilon^{g}\left(r,k\right)\leftarrow 0;\upsilon^{p}\left(r,k\right)\leftarrow 0;k=1\ldots n,\;r=1\ldots N_{b}$$

Initialize the ensemble sets across the samples as follows:

$$\sigma^{g}\left(r,k\right)\leftarrow \emptyset ;\sigma^{p}\left(r,k\right)\leftarrow \emptyset ;k=1\ldots n,\;r=1\ldots N_{b}$$

where *N*_*b*_ represents the number of independent realizations.

Choose a classification technique (e.g. linear discriminant analysis).

**Step 1**: Set *r*←1
Partition the given samples *X*_*mxn*_ into training *V*_*mxk*2_ and test *U*_*mxk*1_ sets such that *k*_1_ *+k*_2_ = *n* and *k*_2_>>*k*_1_.
**Step 2**: Choose a pair of molecules (*i*,*j*),*i*,*j* = 1…m,*i*≠*j*.
**Step 3**: Generate the classifier using the training set for the pair (*i*,*j*).
  - If the *k* ^*th*^ test sample is voted as gingivitis then increment the vote *υ* ^*g*^(*r*,*k*)←*υ* ^*g*^(*r*,*k*)+1. Store the pair (*i*,*j*) in the ensemble set *σ* ^*g*^(*r*,*k*)←Ø.
  - If the *k* ^*th*^ test sample is voted as periodontitis then increment the vote *υ* ^*p*^(*r*,*k*)←*υ* ^*p*^(*r*,*k*)+1. Store the pair (*i*,*j*) in the ensemble set *σ* ^*p*^(*r*,*k*)←Ø.
**Step 4**: Repeat Steps 2 and 3 for each pair of genes (*i*,*j*), *i*,*j* = 1…m,*i*≠*j*.
**Step 5**: Repeat Steps 2–4 using the next window of *k*_1_ samples as test set.
**Step 6**: Determine the classification labels λ*(*k*) of the *k* ^*th*^sample by majority voting as follows
  $$\lambda^{*}\left(k\right)=-1\text{ if}\;\upsilon^{g}\left(r,k\right)>\upsilon^{p}\left(r,k\right)\;\;\;\;\;\;\;\;\text{Gingivitis}$$
  $$+1\text{ if}\;\upsilon^{g}\left(r,k\right)<\upsilon^{p}\left(r,k\right)\;\;\;\;\;\;\;\;\text{Periodontitis}$$
  $$0\text{ if}\;\upsilon^{g}\left(r,k\right)=\upsilon^{p}\left(r,k\right)\;\;\;\;\;\;\;\text{Tie}$$
  If *r<N*_*b*_ then set *r←r*+1, Repeat Steps 1–6 by randomly permuting the columns of *X_mxn_*.
**Step 7**: Proclivity and consensus map across the samples is determined as follows
*Proclivity*: The normalized vote count pair *(G*_*votes*_(*k*), *P*_*votes*_(*k*)) *∈* [0,1] represents the proclivity of the *k* ^*th*^ sample to gingivitis and periodontitis where
  $$G_{votes}\left(k\right)=\frac{1}{N_{b}}\overset{N_{b}}{\underset{r=1}{\sum }}\frac{\upsilon^{g}\left(r,k\right)}{m\left(m-1\right)/2}$$
  $$P_{votes}\left(k\right)=\frac{1}{N_{b}}\overset{N_{b}}{\underset{r=1}{\sum }}\frac{\upsilon^{p}\left(r,k\right)}{m\left(m-1\right)/2}$$
*Consensus Map*: Represents the number of elements that are common between ensemble sets across a pair of samples (*i*,*j*). We define $\sigma^{g*}\left(k\right)=\cap_{r=1}^{N_{b}}\sigma^{g}\left(r,k\right)$ and $\sigma^{p*}\left(k\right)=\cap_{r=1}^{N_{b}}\sigma^{p}\left(r,k\right)$.
  Consensus within the gingivitis group across a pair of samples (*i*,*j*) is given by:
  $$\tau \left(i,j\right)=|\sigma^{g*}\left(i\right)\cap \sigma^{g*}\left(j\right)|\;\left(i,j\right),i,j=1\ldots n_{gin},i\neq j.$$
  Consensus within the periodontitis group across a pair of samples (*i*,*j*) is given by:
  $$\tau \left(i,j\right)=|\sigma^{p*}\left(i\right)\cap \sigma^{p*}\left(j\right)|\;\left(i,j\right),i,j=1\ldots n_{per},i\neq j.$$
  Consensus between the gingivitis and periodontitis groups across samples (*i*,*j*) is given by:
  $$\tau \left(i,j\right)=|\sigma^{g*}\left(i\right)\cap \sigma^{p*}\left(j\right)|\;\left(i,j\right),i=1\ldots n_{gin};\;j=1\ldots n_{per}$$

In the present study, four different classification algorithms (**LDA**: Linear Discriminant Analysis; **QDA**: Quadratic Discriminant Analysis; **NBC**: Naïve Bayes Classifier and **SVM**: Support Vector Machines) were used independently as single classifiers as in Step 1 as a part of the SVA approach (i.e. SVA-LDA, SVA-QDA, SVA-NB, SVA-SVM). Performance of the classification algorithm with and without selective voting is determined assuming the clinical labels of the samples as the ground truth. In Step 1 the given *n* samples were divided into non-overlapping sets with 10 samples each. Each time, a different set is held out as the test data with the remaining samples used as the training data in the classification process. The cross-validation process was independently repeated (*N*_*b*_ = 1000) times by randomly permuting the columns so as to assign different samples to the test and training sets in each independent realization. In contrast to a traditional classifier that uses all markers as features, the proposed approach uses pairs of markers in Step 2 resulting in an ensemble of classifiers that vote on the classification label of a given sample, Step 6. Pairs of markers that vote consistently on a given sample across the (*N*_*b*_ = 1000) realizations were stored in the ensemble set σ^∗^ of that sample in Step 7. Unlike traditional binary classification that deems a sample to either of the disease groups, SVA determines the proclivity of a sample to each of the disease groups given by the normalized vote counts 0 ≤ (*G*_*votes*_, *P*_*votes*_) ≤ 1 in Step 7. Consensus between the ensemble sets of the samples within and between the disease groups reflected by total number of common elements is stored in τ Step 7 and displayed as a heatmap in the present study.

### Classifier Performance and Mismatch Samples

Measures such as (**ACC**: accuracy, **SEN**: sensitivity, **SPC**: specificity) have been used routinely to assess classifier performance. Ensemble vote counts from SVA can result in tie votes when a sample has equal proclivity to each of the disease groups, i.e. *G*_*votes*_ = *P*_*votes*_. Therefore, prior to estimating the performance measures, samples with ties from SVA were randomly assigned labels from one of the classes. While we use the clinical labels of the samples as the ground truth it is important to appreciate that the clinical parameters (BOP, PPD, CAL) may not necessarily capture all intricate aspects of gingivitis and periodontitis. Therefore, samples whose clinical labels and classification labels do not match are termed as mismatch samples and not misclassified samples.

## Results

### Skewed Distributions of Expression Profiles

**Fig 2:**
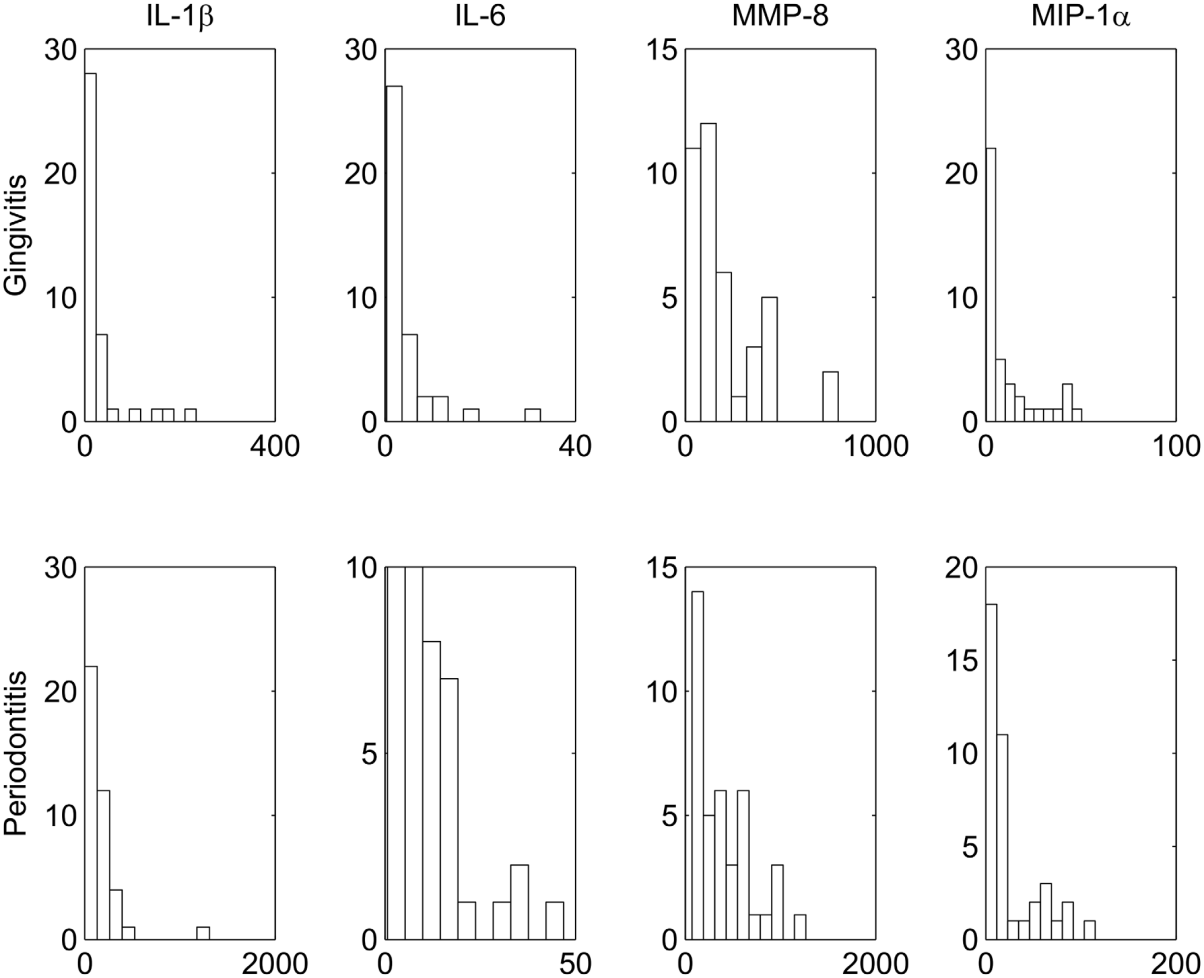
Distribution of the salivary biomarkers. Histograms representing the distribution of molecular expression profiles of (IL-1ß, IL-6, MMP-8, MIP-1α) across the gingivitis (n = 40, top row) and periodontitis (n = 40, bottom row) subjects. The mean and standard deviation of (IL-1ß, IL-6, MMP-8, MIP-1α) across gingivitis were (29.6±49.5, 3.9±5.9, 208.2±194.2, 10.9±14.5) whereas those across periodontitis were (157.6±217, 12.1±10.2, 397.9±302.1, 24.4±29.8).

Clinical criteria (BOP, PPD) with published thresholds are traditionally used to classify a patient either as gingivitis or periodontitis. However, (%BOP, %PPD) across the clinically labelled gingivitis (n = 40) and periodontitis (n = 40) samples revealed marked variations in the magnitude of these parameters within each of the groups. Expression profiles of the salivary biomarkers (IL-1ß, IL-6, MMP-8, MIP-1α) were positively skewed accompanied by large standard deviations and considerable overlap between the disease groups (Fig 2). These findings may indicate the absence of an obvious fixed threshold separating these two disease groups and also the presence of considerable variability within each of the disease groups.

### Performance Measures

Performance measures (ACC, SEN, SPC) estimated with the SVA approach and traditional single classifier approach using four different classification algorithms (LDA, QDA, NBC, SVM) across (*N*_*b*_ = 1000) independent realizations and leave-10-out cross-validation assuming clinical labels as ground truth were comparable (Table 1). Mismatch samples (i.e. classification labels and clinical labels did not match) across a majority of the (*N*_*b*_ = 1000) independent runs and across the four classification algorithms in the SVA approach consisted of 8 gingivitis (4, 15, 17, 18, 23, 25, 28, 38) and 9 periodontitis (41, 47, 51, 62, 64, 67, 70, 72, 78) samples. Three of the mismatch samples (41, 47, 51) were smokers. Thus smoking did not appear to be the primary driving factor behind the mismatch periodontitis samples.

**Table 1:** Classification Performance Metrics with Clinical Labels as Ground Truth.

**SVA Approach**	**Accuracy**	**Sensitivity**	**Specificity**
*SVA-LDA*	0.76±0.02	0.75±0.02	0.77±0.03
*SVA-QDA*	0.76±0.02	0.77±0.02	0.75±0.03
*SVA-NB*	0.76±0.01	0.77±0.02	0.75±0.02
*SVA-SVM*	0.76±0.02	0.73±0.02	0.79±0.03
**Traditional Single Classifier**	**Accuracy**	**Sensitivity**	**Specificity**
*LDA*	0.78±0.01	0.78±0.02	0.78±0.02
*QDA*	0.76±0.02	0.80±0.03	0.72±0.02
*NB*	0.76±0.01	0.77±0.01	0.75±0.01
*SVM*	0.75±0.02	0.74±0.03	0.77±0.02

### Consensus Maps

**Fig 3:**
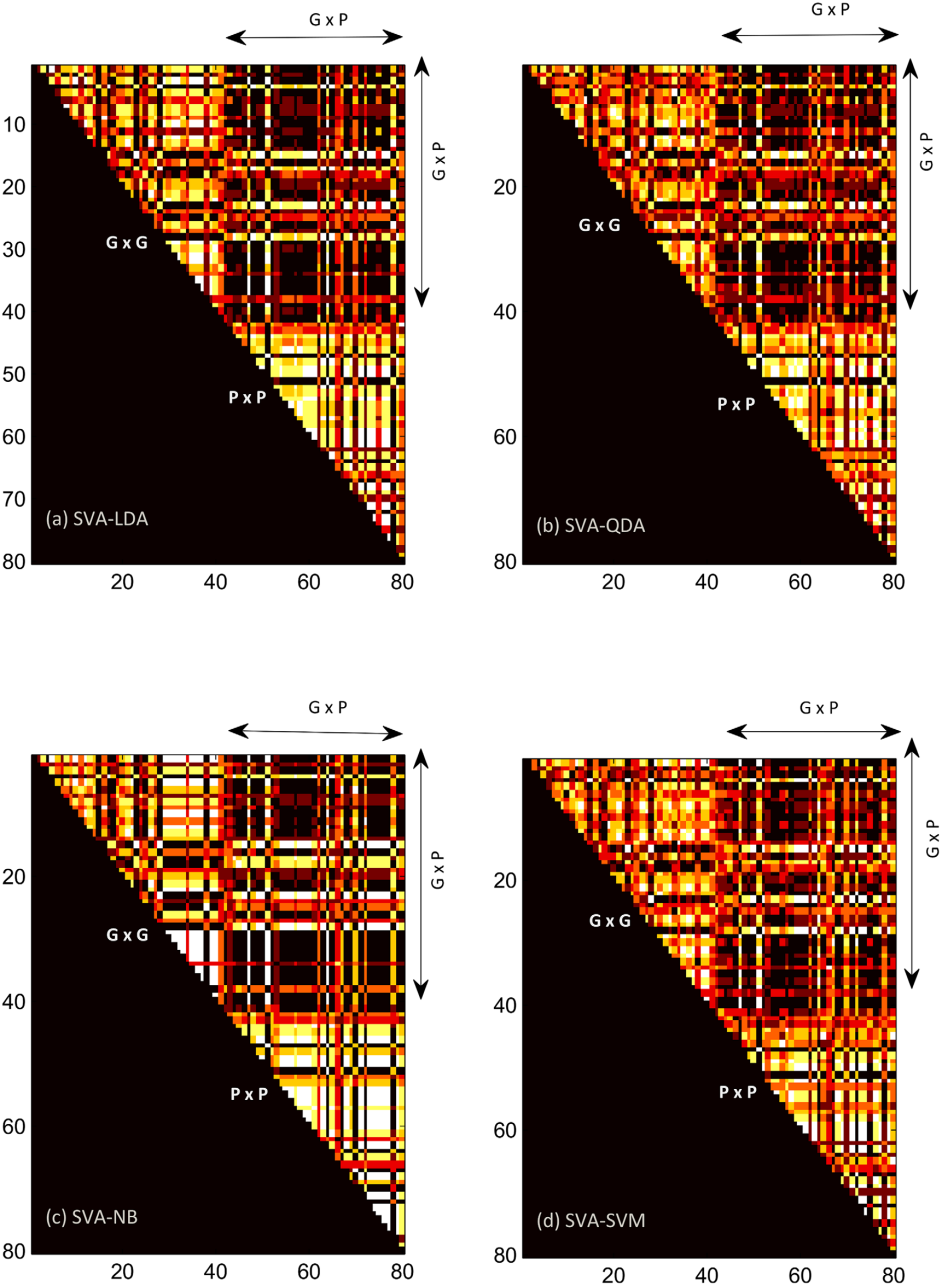
Heatmap Visualization. Heatmap visualization of the consensus map (τ) representing the consensus between the ensemble sets of the gingivitis (1…40) and periodontitis (41…80) samples. Significant overlap is represented by bright color and absence of overlap by dark color. Heatmap for the four different classification techniques (SVA-LDA, SVA-QDA, SVA-NB, SVA-SVM) are enclosed in the subplots (a-d) respectively. In each subplot, there are three distinct regions (**G x G, P x P, G x P**) corresponding to overlap within the gingivitis samples (triangle), within the periodontitis samples (triangle) and between the gingivitis and periodontitis samples (square). The misclassified gingivitis (4, 15, 17, 18, 23, 25, 28, 38) and periodontitis (41, 47, 51, 62, 64, 67, 70, 72, 78) samples are accompanied by pronounced dark streaks in each of the subplots.

Consensus map (τ) representing the magnitude of overlap in the ensemble sets between the samples in the two disease groups is shown in Fig 3. Since the consensus map is symmetric by definition only the upper-triangular part is shown. The consensus map consists of three distinct regions corresponding to consensus within the gingivitis and periodontitis groups represented by the triangular regions **P x P** and **G x G** in Fig 3. In addition, there is also a square region (**G x P**) that represents potential consensus in the ensemble sets between the gingivitis and periodontitis samples. Notably, pronounced dark streaks were observed across the mismatch gingivitis (4, 15, 17, 18, 23, 25, 28, 38) and mismatch periodontitis (41, 47, 51, 62, 64, 67, 70, 72, 78) and the rest of the samples within the respective disease groups in the P x P and G x G regions. While the mismatch samples exhibited lack of consensus within their respective groups, a subset of these samples also exhibited marked consensus between the disease groups accompanied by marked transitions from dark streaks in G x G and P x P to bright streaks in G x P. This subset included gingivitis samples (4, 15, 17, 23, 28) indicating some of the gingivitis samples may share similarity to periodontitis. More importantly, these patterns were consistently observed across the four different classification algorithms (SVA-LDA, SVA-QDA, SVA-NB and SVA-SVM), Fig 3.

### Proclivity

**Fig 4:**
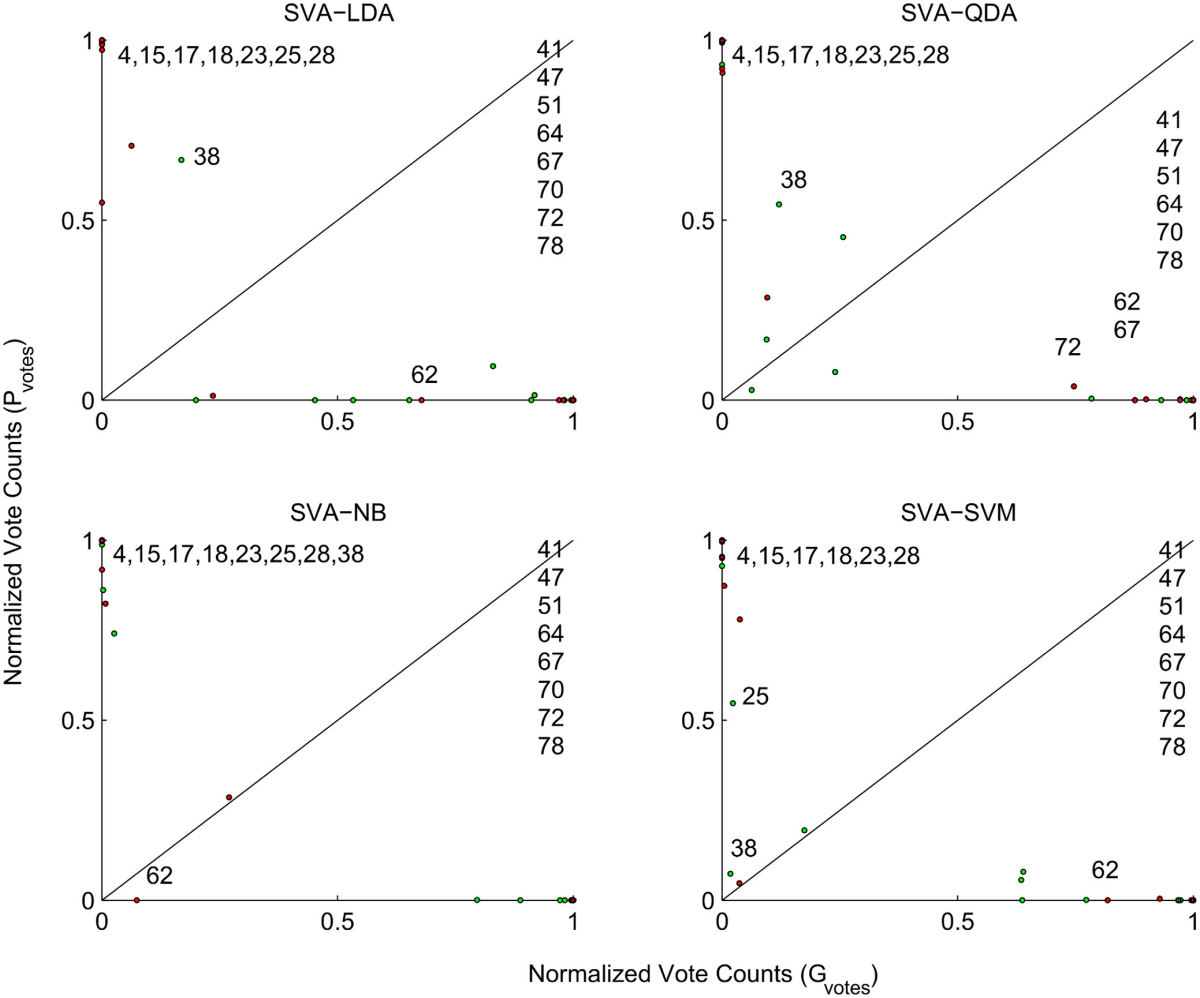
Proclivity (*G*_*votes*_ **,** *P*_*votes*_) of the 80 samples to gingivitis and periodontitis groups obtained using the proposed ensemble selective voting approach is shown. The diagonal (*G*_*votes*_ *= P*_*votes*_) represents the line of separation between the two groups with shift towards abscissa and ordinate representing increasing proclivity to gingivitis and periodontitis respectively. Gingivitis (4, 15, 17, 18, 23, 25, 28, 38, green) and periodontitis (41, 47, 51, 62, 64, 67, 70, 72, 78, red) samples whose clinical and class labels did not march across (SVA-LDA, SVA-QDA, SVA-NB, SVA-SVM) are identified in each of the subplots.

Proclivity of the samples to the gingivitis and periodontitis groups from their normalized vote counts (*G*_*votes*_, *P*_*votes*_) obtained using SVA and the four classification algorithms (SVA-LDA, SVA-QDA, SVA-NB and SVA-SVM) exhibited considerable variation across the samples as shown by the scatter plots (Fig 4). Since (0 ≤ *G*_*votes*_, *P*_*votes*_ ≤ 1) each subplot represents a unit square. The line of separation between gingivitis and periodontitis is represented by the diagonal with samples on the diagonal having equal proclivity to each of these groups i.e. (*G*_*votes*_ *= P*_*votes*_) Shift from the diagonal towards the abscissa and ordinate represent increasing proclivity to gingivitis and periodontitis respectively. While a majority of the mismatch samples exhibited similar proclivities irrespective of the choice of the classification algorithm (SVA-LDA, SVA-QDA, SVA-NB, and SVA-SVM), Fig 4, a few of them (gingivitis: 25, 38) and (periodontitis: 62, 67, 72) were sensitive to the choice of the classification algorithms.

## Discussion

There remain numerous questions regarding the inherent risk of individuals to develop periodontitis, with extensive population variation in genetics and environmental contribution to disease that impact, time of onset, rapidity of progression, and extent of the disease throughout the mouth, combining to dictate the overall severity of the disease [9]. Demographic factors, genetic factors, medical history and risk-factors such as smoking have also been attributed to patient specific variations in gingivitis and periodontitis. These diverse factors in turn warrant developing novel approaches that provide insights into patient-specific variations within disease groups.

A substantial number of investigations over many decades have attempted to catalogue and utilize a range of biologic parameters involved in periodontal disease, focusing on the microbial milieu and the resulting armamentarium of host responses to control this colonization/infection [49]. However, two critical issues that routinely challenge the interpretation of these results is the lack of a clear understanding of the within subject variability and an ability to clearly differentiate the parameters of destructive inflammatory responses in the periodontium. The general strategy has been to utilize a “one size fits all” approach and create a fixed threshold to minimize overlap between clinical categories. In contrast to “biologically homogenizing” the samples within the clinical categories, the proposed approach (SVA) reveals within group heterogeneity of biologic responses as they relate to current clinical presentation from salivary molecular profiles. Thus these findings could be important with respect to personalized medical and dental care in that clinical decisions within this field will rely on recognizing the heterogeneity of patients and understanding patient-specific variations for improving health [58].

The underlying biology of gingivitis and periodontitis involves inflammation, connective tissue destruction and bone remodeling. Biomarkers reflective of these three processes [9, 59] in conjunction with sophisticated classification techniques will enable determining their diagnostic effectiveness and validity. Recent data from our lab and others indicate that salivary concentrations of IL-6, IL-8, albumin, calprotectin, PGE_2_, MMP-8, and MIP-1α are elevated in patients who have gingivitis.[10, 60] Accordingly, these salivary analytes appear to serve as biomarkers of gingivitis, coupled with existing literature on biomarkers of periodontitis [52, 59–63], may help to discriminate those patients who are transitioning from health to gingivitis and on further to periodontitis. Moreover, these salivary markers showed that at least 40% of patients who have gingivitis do not return to biological health within 30 days after standard treatment for gingivitis, even though clinical measures of their gingiva and periodontium indicate a return to clinical health; thus emphasizing the discrepancy between clinical and biological health [10]. Also, our current findings and numerous prior studies have clearly identified important limitations in existing approaches for periodontal disease assessment.

In this vein, we used these putative salivary biomarkers of gingivitis and periodontitis and SVA to evaluate variations in the biological presentations of a cohort of 80 gingivitis and periodontitis patients. Subsequently, we identified a subpopulation of approximately 20% in the cohort that was different biologically from the majority. This was confirmed using four different classification techniques. However, in contrast to traditional classification, SVA approach enabled the markers to vary across the subjects, within and between groups revealing patient-specific variations. As such, this report may begin addressing a fundamental question in periodontology, that is, within the clinical heterogeneity of gingivitis, what patients are at greatest risk for progressing to destructive disease? However, the lack of temporal evaluation of the response to therapy or incidence of transition from gingivitis to periodontitis certainly limits our ability to address this question within the framework of the current study. An additional limitation of the study was that a targeted group of biomarkers were included primarily related to historical evidence of their ability to differentiate periodontitis from health [17, 62, 64]. A subsequent prospective longitudinal experimental design in conjunction with a broader panel of biomarkers [65, 66] would be necessary to demonstrate the prognostic ability of salivary biomarkers for identifying gingivitis patients at greatest risk for progressing to periodontitis. Finally, a more detailed study that explicitly accommodates the various factors that can contribute to patient-specific variations may be necessary to assess the individual contributions of these factors. The findings reported here could help jumpstart the development of personalized decisions in periodontal disease diagnosis and therapy.
